# Resilience, social support, and participation among adolescents in conflict situations

**DOI:** 10.3389/fpsyg.2026.1774403

**Published:** 2026-08-04

**Authors:** Anat Golos, Naomi Weintraub, Yael Lavie-Pitaro, Miri Tal-Saban

**Affiliations:** School of Occupational Therapy, Faculty of Medicine, The Hebrew University of Jerusalem, Jerusalem, Israel

**Keywords:** conflicts, engagement in activities, resilience, social support, youth

## Abstract

**Introduction:**

Conflicts, such as the October 2023 Israel-Gaza war, constitute stressors that may affect individuals’ daily participation and resilience, particularly among adolescents. The socio-ecological resilience perspective emphasizes that effects of adversity are not uniform, but vary according to individuals’ access to, and capacity to utilize, contextual resources. However, research in this area is scarce. This study examined the contribution of social support (family and friends), and participation in daily activities at school, home and the community, to adolescents’ resilience in the context of war.

**Methods:**

Using a cross-sectional, correlational design, 209 Israeli 10th–11th graders completed measures of social support, daily participation and resilience.

**Results:**

After controlling for gender, regression analysis revealed that family and friends’ support, along with perceived importance of school participation, contributed significantly and uniquely to adolescents’ resilience. The final model explained a total of 37.3% of the variance in resilience.

**Discussion:**

The findings support the socio-ecological resilience perspective, highlighting the potential protective role of family, friends and school participation in shaping adolescents’ resilience during war. The results may guide future research in this area, and offer practical directions for developing educational and health programs aimed at strengthening adolescents’ resilience in adverse situations.

## Introduction

Conflict is frequently considered a significant stressor with far-reaching personal consequences for daily functioning, triggering anxiety, depression, and post-traumatic stress disorder (PTSD; Klochko, 2024; Osokina et al., 2023). The terrorist attack on Israel on October 7, 2023, and the subsequent war represent a complex and prolonged situation characterized by substantial stress, tension, and trauma, affecting large segments of the population (Marciano et al., 2024; Sarid et al., 2025). Adolescents, in particular, represent a group at heightened risk for these adverse effects and are therefore the focus of the present study.

Adolescence is defined as a transitional period between childhood and adulthood. Although the exact age range of adolescence is not universally agreed upon, it is generally considered to span ages 10 or 11–19 or 21 (Hagan et al., 2017; World Health Organization, 2007). This period is regarded as complex and dynamic, characterized by substantial biological and cognitive changes (Blakemore and Mills, 2014; Hagan et al., 2017), often accompanied by increased emotional reactivity and identity exploration (Syed and McLean, 2017). These developmental processes may heighten adolescents’ vulnerability to both everyday stressors and acute adversities, such as those associated with war (Klochko, 2024; Osokina et al., 2023). Exposure to such stressors may activate physiological responses that compromise executive functioning and self-regulation, which are essential for adaptive functioning in challenging contexts (Betancourt, 2015; Dias and Cadime, 2017).

At the same time, adolescence is also characterized by increased autonomy from parents and a growing reliance on peer relationships as primary sources of emotional support and belonging (Lam et al., 2014). These meaningful relationships often play a significant role in adolescents’ coping process during this critical period and in the development of resilience (Höltge et al., 2021; Ungar et al., 2013).

During the current war, adolescents in Israel, have been exposed to various stressful events. They experienced disruptions to their daily routines, such as evacuation or displacement from their homes and communities, interruptions to their schooling and extracurricular activities, and separation from peers. Some have also been exposed to terrorist attacks and missile strikes, leading to physical or emotional harm to themselves, family members, or acquaintances. Consequently, their patterns of participation in daily activities may have changed or been restricted, potentially impacting their well-being and resilience.

Resilience is defined as the process of adapting to, coping with, and recovering from, personal or familial crises, serious illnesses, or other stressful or traumatic events (American Psychological Association, 2014; Fino et al., 2020; Lee et al., 2014; Olsson et al., 2003). Consistent with Rutter’s conceptualization (Rutter, 1987, 2006), resilience is not a fixed trait but a dynamic process that is expressed in the presence of adversity. Building on the socio-ecological resilience perspective (Ungar et al., 2013) and specifically, the Differential Impact Theory (DIT; Ungar, 2018), the effects of risk and protective factors may vary across individuals depending on their access to, and capacity to utilize, contextual resources. For the purposes of the current study, DIT was applied in a limited sense, specifically in relation to differential access to and utilization of contextual resources across adolescents, such as social support and opportunities for participation in daily activities. While the broader DIT framework also emphasizes variability in the level and nature of adversity exposure, this aspect was beyond the scope of the current study and was not directly examined. Rather, the present study focused on how differences in access to and use of contextual resources may contribute to resilience among adolescents within the broader context of war.

From this perspective, resilience is shaped by the availability, accessibility, and meaningfulness of resources within an individual’s environment, as well as by individuals’ ability to access these resources. Hence, the same environmental conditions may lead to different outcomes across adolescents, depending on how resources are distributed and experienced. Moreover, studies have shown that resilience level may also be gender-related. Several studies reported that males are more likely to demonstrate higher levels of resilience compared to females (Fayyad et al., 2017; Gök and Koğar, 2021; Guilera et al., 2015).

One factor that may provide adolescents with opportunities to access and benefit from available protective resources, and that has been associated with resilience, is participation in daily activities (Fayyad et al., 2017; Oliver et al., 2006). The International Classification of Functioning, Disability and Health (ICF) framework of the World Health Organization (WHO; World Health Organization, 2007) defines participation as involvement in everyday activities across all life domains, including self-care (e.g., bathing, dressing, cooking), education, work, play, and leisure (e.g., hobbies, sports, socializing with friends). These activities may occur alone or with others and across settings, including school, home, and the community (Bedell et al., 2013). Participation supports the development of competencies, facilitates social role engagement, and promotes a sense of belonging (Imms et al., 2017), all of which may support the development of resilience. Furthermore, participation is a multidimensional construct that includes both an objective aspect (e.g., frequency of participation), and a subjective aspect, encompassing individuals’ perceptions and emotions related to participation (e.g., importance or meaningfulness they attribute to an activity) (Coster et al., 2012).

However, from an occupational science perspective, participation extends beyond mere involvement or frequency of engagement in activities, and also relates to engaging in occupations that hold personal and social meaning within specific environmental and cultural contexts (Hammell, 2004; Wilcock, 1999). Meaningful participation may foster a sense of competency, agency, identity, and social connectedness, and promote a sense of belonging (Imms et al., 2017; Oliver et al., 2006), which could be considered important protective processes during periods of adversity and forced disruption, such as war. In such contexts, participation in meaningful activities may help preserve routines, social roles, and a sense of continuity, despite ongoing stress and uncertainty, that may support the development of resilience (Nizzero et al., 2017). This perspective aligns with socio-ecological approaches to resilience, including Ungar’s Differential Impact Theory (Ungar, 2018), which proposes that resilience is shaped not only by individuals’ characteristics, but also by their ability to access and meaningfully utilize protective resources within their environments.

Nevertheless, it should be noted that the relationship between participation and resilience may be bidirectional. On the one hand, coping abilities and resilience may support adolescents’ participation in social and daily activities (Kim and Park, 2023; Oliver et al., 2006). On the other hand, aligned with the socio-ecological resilience perspective (Ungar et al., 2013), meaningful participation in daily activities may serve as a protective factor and enhance resilience by supporting a sense of competence and agency, both of which are essential for building resilience (Oliver et al., 2006). However, currently, this relationship has limited empirical evidence among adolescents (Fayyad et al., 2017; Oliver et al., 2006), particularly in the context of war.

The ICF framework further highlights the role of environmental factors in shaping participation (World Health Organization, 2007). Among these, social support constitutes a key contextual resource, particularly during adolescence (Oliver et al., 2006). Social support has various definitions, but is commonly defined as the availability or perception of emotional, informational, and instrumental resources provided by one’s social network, which facilitate coping with stress (Song and Zhang, 2023). Importantly, consistent with Rutter’s view conceptualization of resilience as a dynamic process involving protective mechanisms (Rutter, 1987, 2006), resilience reflects the ability to resist environmental risk experiences and overcome stress in the presence of adversity. It is shaped through interactions with the environment. Hence, social support may function as a protective factor enabling adolescents to cope with and adapt to challenging conditions.

The present study conceptualizes social support in accordance with the Multidimensional Scale of Perceived Social Support (Zimet et al., 1988), which distinguishes among three primary sources: family, friends, and significant others. Family support may provide stability, peer support may foster belonging and identity formation, and support from significant others may reflect close, trust-based relationships that extend beyond these domains. The relative contribution of each source may therefore differ across individuals and circumstances. Empirical evidence indicates that perceived social support from these sources is associated with both greater participation and enhanced resilience (Oliver et al., 2006; Ozbay et al., 2007; Sippel et al., 2015; Tollit et al., 2015; Uysal et al., 2022). Supportive relationships may enable adolescents to maintain engagement in daily activities under conditions of stress.

Taken together, the integration of the socio-ecological resilience perspective with the ICF framework (World Health Organization, 2007) suggests that adolescents’ resilience in contexts of war may be shaped by the interplay between the social support available to them and their participation in meaningful daily activities. While several studies have examined the characteristics of adolescents’ resilience during wars (Fayyad et al., 2017; Osokina et al., 2023; Uysal et al., 2022), no studies have been found that examined the unique contribution of adolescents’ participation in daily activities across settings alongside social support to their resilience during wartime.

The overarching aim of the present study was to examine the unique contribution of social support across family and friends, as well as participation in daily activities, at school and in the home and community, to adolescents’ resilience in the context of war. Resilience was operationally defined only based on the Individual domain of the Adolescent Resilience Questionnaire (ARQ; Gartland et al., 2011). This study aimed at answering the following questions: (1) Is there an association between social support, participation in daily activities and resilience among high school adolescents during the war?; and (2) What is the contribution of perceived social support to the variance in adolescents’ resilience, and does participation have a unique contribution to resilience beyond social support? We hypothesized that perceived social support (defined in terms of subjectively assessed social support from family, friends, and significant others) will have a significant contribution to the variance in adolescents’ resilience and that participation in daily activities will account for additional variance in resilience.

## Methods and materials

### Study design and participants

The study employed a cross-sectional, correlational, and comparative design, based on a convenience sample. The principals of seven public general-education high schools in various regions in Israel were approached, of whom five consented to participate. In these schools, 233 adolescents in 10th and 11th-grades, whose parents provided written consent and who themselves gave oral consent to participate in the study, were asked to fill out the questionnaires of the study battery; 24 adolescents (10.3%) declined. Thus, the study population consisted of 209 adolescents. Sample size was determined using G*Power software (Faul et al., 2009). Based on a previous study that focused on the correlation between resilience and social support among children (Folayan et al., 2020), with a significance level of 5%, test power of 70%, the required sample size was 208 participants. See Table 1 for the distribution of socio-demographic background variables.

**TABLE 1:** Distribution of socio-demographic background variables (*N* = 209).

Variable	*n* (%)
Gender	
Boys	120 (57.4)
Girls	89 (42.6)
Grade	
10th grade	120 (57.4)
11th grade	89 (42.6)
Parental marital status1	
Married	173 (83.6)
Single	34 (16.4)

^1^Two participants did not report parental marital status.

### Procedure

The study received approval from the Ministry of Education (No. 11836) and from the Ethics Committee of the Hebrew University (No. 23012024). High school principals were contacted and provided with information about the study. Five principals agreed to participate, and they distributed letters to parents via homeroom teachers to obtain written consent for their children’s participation. Data collection took place between March and June 2024, carried out by a doctoral student who visited the classrooms during school hours. She explained the purpose of the study, and that the questionnaires were completed anonymously. Adolescents who provided oral consent to participate completed the questionnaires in approximately 30 min.

### Measures

#### Socio-demographic questionnaire

This self-report questionnaire was developed for the current study to gather demographic information; i.e., grade level, gender, parental marital status.

#### Adolescents’ Participation Questionnaire (AdPQ)

The AdPQ assesses participation characteristics of adolescents in activities across school, home, and community settings (Lavie-Pitaro et al., 2025). It includes 28 typical adolescent activities (e.g., learning, leisure, and volunteering), 6 of which relate to school-based settings and 22 to home and community settings. Originally, each item is scored on three scales – Frequency of participation, Importance of the activity to the student, and Satisfaction with the activity. Both Frequency and Importance are rated on a scale from 1 (”Not at all”) to 4 (”Always” or “Very much,” respectively). In the current study, we replaced the Satisfaction scale of the AdPQ with a new scale relevant to the current study: “Changes in Participation Frequency” due to the war. This change occurred due to time constraints imposed by the schools for data collection. The Adolescents were asked to report the extent of change in their participation in each of the activities, compared to before the war (rated on a scale of 1 = “Participate less” and 0 = “Participate more” or “Same as usual”). A higher score reflects a decline in participation. Average scores were calculated for each scale separately, and no total score was used. The questionnaire was previously found to be valid and reliable (overall internal consistency α = 0.74, test-retest reliability ICC = 0.42–0.92). In the current study, overall internal consistency was very good (α = 0.88).

#### Multidimensional Scale of Perceived Social Support (MSPSS)

The MSPSS assesses adolescents’ perceived social support from three sources: family, friends, and significant others (Zimet et al., 1988). The original version includes 12 items, 4 for each source of support. However, in the current study, due to ambiguity regarding the term “significant others,” only the items measuring family and friends support were included. Adolescents rated how well each item reflected their experience on a 7-point scale (1 = “Very strongly disagree” to 7 = “Very strongly agree”). Separate average scores are calculated for each support source, as well as a total support score. Higher scores reflect higher perceived social support. The original MSPSS was found to be valid and reliable (α = 0.88). In the current study, internal consistency was excellent for both family (α = 0.93) and friends support (α = 0.93).

#### Adolescent Resilience Questionnaire (ARQ)

The ARQ assesses adolescents’ resilience across various contexts: individual, family, friends, school, and community (Gartland et al., 2011). The questionnaire includes 88 items rated on a 5-point scale (1 = “Never” to 5 = “Always”). The total score is calculated as the sum of all items, with higher scores indicating higher resilience. The ARQ has demonstrated good internal consistency (α = 0.70–0.90) and convergent validity. In this study, only the Individual domain was used, in accordance with a request from the Ministry of Education to reduce participant burden. This domain includes five factors: Confidence, Emotional Insight, Negative Cognition, Social Skills, and Empathy/Tolerance. Cronbach’s alpha values in the current sample were α = 0.78 for Confidence, α = 0.68 for Emotional Insight, α = 0.84 for Negative Cognition, α = 0.79 for Social Skills, and α = 0.49 for Empathy/Tolerance. The overall internal consistency of the Individual domain was very good (α = 0.90).

### Data analysis

Data analysis was conducted using SPSS version 31 (IBM Corp, 2022), with a significance level set at 0.05. Descriptive statistics (e.g., means, standard deviations, and percentages) were used to describe the sample and variable distributions. Internal consistency of the research measures in the current study sample was calculated using Cronbach’s alpha. Distribution of the data (based on skewness and kurtosis tests) showed that participation in school and resilience scores were normally distributed, whereas participation in home and community activities, changes in participation, and social-support scores were not. Relationships between variables were assessed using Pearson or Spearman correlations, as appropriate. False discovery rate (FDR) correction for multiple comparisons was applied using the Benjamini-Hochberg procedure (Benjamini and Hochberg, 1995). Finally, a mixed hierarchical-stepwise linear regression analysis was conducted to explain variance in resilience. To control for possible gender effect, it was entered hierarchically in Step 1. Social-support variables were forced into the model in Step 2, followed by participation variables in Step 3 using a stepwise method. This enabled to examine which, if any, of the participation variables significantly contributed to explaining resilience beyond the variance already accounted for by gender and social support.

## Results

### Descriptive statistics of the study variables

Table 2 presents the descriptive statistics, including means and standard deviations, for the primary study variables: social support, participation, and resilience. Regarding social support (rated on a 1–7 scale), participants reported relatively high levels of support of family and friends. In terms of participation (rated on a 1–4 scale), adolescents reported moderate-to-high levels of frequency and importance across settings. The reported decline in participation due to the war (rated on a 0–1 scale, where higher scores reflect a greater decrease) was relatively low for both school activities and home and community activities. Finally, the sample demonstrated a mean total individual resilience score of 136.03 (SD = 19.61), alongside the descriptive values for its five corresponding subscales (Confidence, Emotional insight, Negative cognition, Social skills, and Empathy/Tolerance).

**TABLE 2:** Social support, participation in activities, and resilience (*N* = 209).

Variable	M (SD)
Social support	
Family	5.79 (1.44)
Friends	5.54 (1.47)
Participation in school	
Frequency	2.71 (0.58)
Importance	2.80 (0.64)
Participation in home and community activities	
Frequency	2.46 (0.38)
Importance	2.96 (0.51)
Change in participation1	
School activities	0.22 (0.25)
Home and community activities	0.24 (0.20)
Resilience	
Total	136.03 (19.61)
Confidence	30.54 (5.01)
Emotional insight	29.01 (4.63)
Negative cognition	23.61 (6.50)
Social skills	26.08 (5.84)
Empathy/tolerance	26.79 (4.18)

^1^A higher score in change in activities indicates a greater decrease in participation.

### Association between social support, participation and resilience

To address the first research question, correlations between social support, participation, and resilience were examined (see Table 3). Moderate and highly significant positive correlations were found between both sources of social support (family and friends) and the total resilience score, as well as all the ARQ’s sub-scales, indicating that higher support is associated with higher resilience. However, the most strongly associated subscales were Confidence and Emotional insight subscales. Moderate and highly significant positive correlations were also observed between both sources of support and Social skills.

**TABLE 3:** Spearman and pearson correlations between social support, participation and resilience (*N* = 209).

Variable	Resilience					
	Total	Confidence	Emotional insight	Negative cognition	Social skills	Empathy/tolerance
Social support1						
Family support	0.428***	0.466***	0.456***	0.178*	0.338***	0.219**
Friend support	0.421***	0.347***	0.387***	0.226**	0.373***	0.291***
Participation in school activities2						
Frequency	0.280***	0.381***	0.303***	0.069	0.266***	0.040
Importance	0.288***	0.351***	0.276***	0.126	0.204**	0.144
Participation in Home and community activities1						
Frequency	0.102	0.197**	0.177*	−0.044	0.032	0.035
Importance	0.082	0.155*	0.175*	−0.079	0.041	0.063
Changes in participation1						
School activities	−0.210**	−0.207**	−0.175*	−0.160*	−0.136	−0.121
Home and community activities	−0.122	−0.117	−0.149*	−0.039	−0.097	−0.107

^1^Spearman correlation;

^2^Pearson correlation; the correlations in the table are after FDR; A higher score in change in participation indicates a greater decrease in participation;

*p < 0.05; **p < 0.01; ***p < 0.001.

Additionally, low but significant positive correlations were found between both the frequency and importance of participation in school activities and total resilience, as well as most of the resilience sub-scales (except for Negative cognition and Empathy/Tolerance), indicating that higher frequency and importance of participation in school are associated with greater overall resilience. Furthermore, a significant negative correlation was observed between changes in school participation and total resilience. Because a higher score in this scale indicates a greater decrease in participation, this finding suggests that a larger drop in school participation frequency is linked to lower resilience. In contrast, no significant correlations were found between home and community participation (frequency, importance, or changes) and total resilience.

To address the second research question and examine the relative contribution of social support and participation in explaining the variance in resilience (while controlling for gender), a mixed hierarchical-stepwise linear regression analysis was conducted (Table 4). The analysis yielded a significant final model. To isolate the specific contribution of gender, it was entered in the first step to control for its baseline effect, and was found to account for 12.5% of the variance in resilience (*p* < 0.001). In Step 2, social support variables (friend and family support) were entered, and were found to uniquely and significantly contribute additional 22.6% to the explained variance of resilience (ΔR^2^ = 0.225, *p* < 0.001). As expected, higher levels of support from both family and friends were associated with greater resilience. Finally, the stepwise entry of the participation variables in Step 3 revealed that only the importance of participation in school activities significantly contributed to the model. It added a further 2.2% to the explained variance (ΔR^2^ = 0.022, *p* = 0.009), suggesting that a higher perceived importance of school activities accounted for an increased resilience. None of the other participation variables met the statistical criteria for inclusion. All together, the final model explained a total of 37.3% of the variance in resilience.

**TABLE 4:** Hierarchical stepwise linear regression explaining variance in resilience^1^.

Step	Variables	B	SE	β	Δ R^2^	R^2^
1	Constant	127.984	1.948	0.354***	0.125***	0.125***
	Gender^1^	14.008	2.571			
			
2	Constant	88.261	5.012	0.321***	0.226**	0.351***
	Gender^1^	12.713	2.245			
	Friend support	3.735	0.945	0.280***		
	Family support	3.412	0.966	0.251***		
			
3	Constant	77.908	6.276		0.022**	0.373***
	Gender^1^	11.728	2.242	0.296***		
	Friend support	3.641	0.932	0.273***		
	Family support	3.106	0.959	0.229***		
	Importance of participation in school activities	4.715	1.764	0.153**		
			

^1^Gender – 0 = Girls; 1 = Boys. **p < 0.01; ***p < 0.001.

## Discussion

Following the events of the October 2023 war, many adolescents in Israel were exposed to various stressful events, including displacement from their homes and communities, separation from their peers, and disruption to their daily routines, including their schooling and extracurricular activities. Hence, their patterns of participation in everyday activities were altered. Additionally, many adolescents or their family members experienced physical or emotional harm. The few existing studies on this topic among adolescents (Fayyad et al., 2017; Uysal et al., 2022) have suggested that both social support and participation in daily activities may enhance adolescents’ resilience by promoting social connectedness and coping capacities, particularly during periods of adversity.

Consistent with the socio-ecological resilience perspective (Ungar et al., 2013), resources such as social support and opportunities for participation may function as protective factors. However, their contribution to resilience may depend on adolescents’ access to these resources, as well as on how meaningful they are to them. Accordingly, the overall aim of the present study was to examine whether social support and participation in daily activities contributed to explaining the variance in adolescents’ resilience. It is important to note that data collection took place approximately 6 months after the onset of the war, allowing adolescents sufficient time to experience and report not only their current participation patterns, but also perceived changes in participation following the onset of the war.

### Resilience

In examining the resilience levels of the study’s sample, adolescents reported resilience levels similar to adolescents who were not exposed to war-related situations such as in Spain (Guilera et al., 2015) or Nepal (Singh et al., 2019). Given that the students had been exposed to the war for 6 months at the time of data collection, this finding is somewhat surprising. One possible explanation is that adolescents who had access to protective factors, such as social support and participation in daily activities, may have been better equipped to cope with war-related stress (Hashemi and Mahmoudzadeh, 2025; Kimhi et al., 2010). However, given the cross-sectional design of the study, no causal or developmental inferences can be drawn from these findings.

### Contribution of social support and participation in daily activities

This study’s findings showed significant correlations between both family and social support and the adolescents’ resilience in all subscales. Moreover, both types of support had a significant and unique contribution in explaining the variance in resilience, and together contributed 22.6% to the explained variance. As expected, higher levels of support from both family and friends were associated with greater resilience. These findings are congruent with previous research underscoring the role of social support in fostering resilience (Betancourt and Khan, 2008; Çiçek, 2021; McLaughlin et al., 2021; Tollit et al., 2015), as support provides psychological resources for stress management (Cohen, 2004) and development of coping mechanisms (Ozbay et al., 2007; Sippel et al., 2015; Tollit et al., 2015).

Additionally, this study’s results indicated that for the adolescents who were exposed to war, both the frequency and importance of participation in school activities, as well as change in these activities were associated with resilience, as opposed to participation in home and community activities. However, only importance of participation in school activities had a unique and significant contribution to the resilience variance. This may stem from the fact that school serves as a stabilizing anchor in adolescents’ lives, contributing to a sense of routine and stability, thereby promoting resilience (Brooks, 2006; Liebenberg et al., 2016; Oberle et al., 2019; Theron, 2016). This premise aligns with occupational science theories suggesting that, in addition to enhancing a sense of competency, agency, and belonging (Imms et al., 2017; Oliver et al., 2006), participation in meaningful school activities may help maintain students’ routines and sense of continuity, despite their stressful and unstable reality, thereby supporting the development of resilience (Nizzero et al., 2017).

A closer examination of the findings showed that, as opposed to the positive associations between school participation and the Confidence, Emotional insight and Social skills sub-sclaes of the ARQ (Gartland et al., 2011), school participation was not significantly correlated with the Negative cognition and Empathy/Tolerance sub-scales. We could not find similar studies examining the association between school participation and the different subscales of the ARQ (Gartland et al., 2011). However, participation in meaningful daily activities has been shown to be associated with adolescents’ development and school and social connectedness (Imms et al., 2017; Too et al., 2022).

Participation in school activities may be more strongly related to resilience dimensions that are behaviorally and socially expressed, such as confidence, emotional insight, and social skills (Ungar, 2018), because these capacities are directly reinforced, and practiced through everyday engagement with others and through successful participation experiences. Thus, adolescents who continued to participate in school activities during the war may have maintained routines, peer interaction, and social relationships, all of which may strengthen confidence and social functioning.

In contrast, negative cognition (e.g., emotional regulation, pessimism) and empathy may represent more internal cognitive-affective processes (Gartland et al., 2011; Guilera et al., 2015) that, perhaps, are less directly shaped by school participation itself. This interpretation is broadly consistent with socio-ecological resilience perspective (Ungar et al., 2013), suggesting that protective factors may contribute differently across resilience domains. From this perspective, enhanced school participation may have been less relevant to the aspects of negative cognition and empathy dimensions of resilience among the adolescents in the present study.

Finally, this study’s findings suggested that participation in home and community activities was weakly associated with resilience, and most often the correlations were not statistically significant. These findings contradict previous studies reporting positive associations between participation in community activities (Oliver et al., 2006), and more specifically, leisure activities (Fayyad et al., 2017) and enhanced resilience. The discrepancy between the findings may be related to differences in the age groups examined or in the measures used compared with those in Fayyad et al.’s (2017) study. Another possible explanation is that, during the war, many home- and community-based activities were disrupted, whereas school frameworks continued to function relatively consistently. Consequently, opportunities for participation in home and community activities may have been less available to adolescents and therefore less able to function as protective resources within the context of the present study. However, the limited research examining participation across different settings and resilience among adolescents warrants further investigation of this hypothesis.

### Strenghts, limitations and recommendations for future research

The primary strength of this study lies in its focus on adolescents, an understudied population that is often characterized by heightened emotional instability and identity exploration. By adopting a person-centered approach, the study enabled adolescents to express their own perceptions of resilience, social support, and participation across multiple contexts, including school, home and community settings.

However, several limitations should be noted. First, the cross-sectional nature of this study, conducted approximately 6 months after the war, prevents the determination of whether higher resilience reflects a consequence of exposure to the war or pre-existing individual differences. Second, the results rely on self-report questionnaires, which may be subject to bias. Furthermore, the assessment of resilience focused primarily on the individual level; therefore, broader systemic levels were not directly captured. In addition, the absence of the satisfaction dimension of participation may affect the understanding of the full experience of subjective participation. Finally, the data-driven selection of the participation variables should be interpreted with caution.

Future studies should adopt longitudinal designs to examine the association of social support, participation and resilience over time. Subsequent studies could also explore the associations between these factors and specific mental health outcomes. To enrich the data, future research should incorporate objective indicators of resilience in broader systematic levels, such as academic achievement or school attendance, and explicitly include school-based support within a broader systemic context. Finally, qualitative studies, focusing on adolescents’ perspectives, including their satisfaction with participation at home and in the community, and social support, would deepen the understanding of the factors contributing to adolescents’ resilience.

### Practical implications

The findings of this study highlight several practical implications for strengthening adolescents’ resilience of in context of war. First, school participation appears to play a central role in supporting adolescent resilience. Therefore, efforts should be directed toward maintaining regular school attendance during times of war and encouraging adolescents’ engagement in meaningful school-based activities. The findings also underscore the importance of sustaining adolescents’ key social support networks, including family members, peers, and school staff. Therefore, it is important to ensure that these social networks are maintained and sustained during periods of crisis or conflict. To this end, families should receive guidance and support during times of crisis in order to enhance their ability to support their adolescents. Maintaining and strengthening these key support networks, may play an important role in fostering and strengthening adolescents’ resilience.

Previous research has shown that school-based programs, particularly multicomponent and cognitive behavioral therapy (CBT)-based interventions, are effective in promoting resilience among at-risk adolescents (Cai et al., 2025; Llistosella et al., 2023). Programs such as Cognitive Behavioral Intervention for Trauma (Stein et al., 2003) and FRIENDS for Life (Maalouf et al., 2020) focus on developing coping, emotional regulation, and problem-solving skills among trauma-exposed youth. The current findings suggest that strengthening school participation and social support networks may further enhance the effectiveness of such interventions among adolescents exposed to war.

## Conclusion

This study examined the contribution of social support across family and friends, as well as participation in daily activities, at school and in the home and community, to adolescents’ resilience in the context of war. Results showed that both social support and school participation contributed to the variance in resilience. These findings align with the Rutter’s conceptualization of resilience as a dynamic process expressed in the presence of adversity (Rutter, 1987, 2006) and with the Differential Impact Perspective (Ungar, 2018), emphasizing variability in access to and utilization of contextual resources.

To our knowledge, this is among the few studies examining how participation across school, home, and community settings, beyond the support they receive from family and friends, may support adolescents’ resilience during conflicts. These results may guide future research in this area and offer practical directions for developing educational and health programs aimed at strengthening adolescents’ resilience in adverse situations, such as war or other conflict situations.

## Data Availability

The datasets presented in this article are not readily available because the dataset is not publicly available due to ethical restrictions and the need to protect participants’ privacy and confidentiality. Requests to access the datasets should be directed to AG, anat.golos@mail.huji.ac.il.

## References

[B1] American Psychological Association (2014). *The Road to Resilience.* Washington, D.C: American Psychological Association.

[B2] Bedell G. Coster W. Law M. Liljenquist K. Kao Y. C. Teplicky R. et al. (2013). Community participation, supports, and barriers of school-age children with and without disabilities. *Arch. Phys. Med. Rehabil.* 94 315–323. 10.1016/j.apmr.2012.09.024 23044364

[B3] Benjamini Y. Hochberg Y. (1995). Controlling the false discovery rate: A practical and powerful approach to multiple testing. *J. R. Stat. Soc. B* 57 289–300. 10.1111/j.2517-6161.1995.tb02031.x

[B4] Betancourt T. S. (2015). The intergenerational effect of war. *JAMA Psychiatry* 72 199–200. 10.1001/jamapsychiatry.2014.2669 25565552

[B5] Betancourt T. S. Khan K. T. (2008). The mental health of children affected by armed conflict: Protective processes and pathways to resilience. *Int. Rev. Psychiatry* 20 317–328. 10.1080/09540260802090363 18569183 PMC2613765

[B6] Blakemore S. J. Mills K. L. (2014). Is adolescence a sensitive period for sociocultural processing? *Annu. Rev. Psychol.* 65 187–207. 10.1146/annurev-psych-010213-115202 24016274

[B7] Brooks J. E. (2006). Strengthening resilience in children and youths: Maximizing opportunities through the schools. *Child Sch.* 28 69–76. 10.1093/cs/28.2.69

[B8] Cai C. Mei Z. Wang Z. Luo S. (2025). School-based interventions for resilience in children and adolescents: A systematic review and meta-analysis of randomized controlled trials. *Front. Psychiatry* 16:1594658. 10.3389/fpsyt.2025.1594658 40458775 PMC12127306

[B9] Çiçek İ (2021). Effect of hope on resilience in adolescents: Social support and social connectedness as mediators. *J. Posit. School Psychol.* 5 136–147. 10.47602/jpsp.v5i2.283

[B10] Cohen S. (2004). Social relationships and health. *Am. Psychol.* 59 676–684. 10.1037/0003-066X.59.8.676 15554821

[B11] Coster W. Law M. Bedell G. Khetani M. Cousins M. Teplicky R. (2012). Development of the participation and environment measure for children and youth: Conceptual basis. *Disabil. Rehabil.* 34 238–246. 10.3109/09638288.2011.603017 21981404

[B12] Dias P. C. Cadime I. (2017). Protective factors and resilience in adolescents: The mediating role of self-regulation. *Psicol. Educ.* 23 37–43. 10.1016/j.pse.2016.09.003

[B13] Faul F. Erdfelder E. Buchner A. Lang A. G. (2009). Statistical power analyses using G*Power 3.1: Tests for correlation and regression analyses. *Behav. Res. Methods* 41 1149–1160. 10.3758/BRM.41.4.1149 19897823

[B14] Fayyad J. Cordahi-Tabet C. Yeretzian J. Salamoun M. Najm C. Karam E. G. (2017). Resilience-promoting factors in war-exposed adolescents: An epidemiologic study. *Eur. Child Adolesc. Psychiatry* 26 191–200. 10.1007/s00787-016-0871-0 27312537

[B15] Fino E. Mema D. Russo P. M. (2020). War trauma exposed refugees and posttraumatic stress disorder: The moderating role of trait resilience. *J. Psychosom. Res.* 129:109905. 10.1016/j.jpsychores.2019.109905 31869693

[B16] Folayan M. O. Oginni O. Arowolo O. El Tantawi M. (2020). Internal consistency and correlationof the adverse childhood experiences, bully victimization, self-esteem, resilience, and social support scales in Nigerian children. *BMC Res. Notes* 13:362. 10.1186/s13104-020-05174-3 32650832 PMC7350627

[B17] Gartland D. Bond L. Olsson C. A. Buzwell S. Sawyer S. M. (2011). Development of a multi-dimensional measure of resilience in adolescents: The adolescent resilience questionnaire. *BMC Med. Res. Methodol.* 11:134. 10.1186/1471-2288-11-134 21970409 PMC3204306

[B18] Gök A. Koğar E. Y. (2021). A meta-analysis study on gender differences in psychologicalresilience levels. *Cyp. Turk. J. Psychiatry Psychol.* 3 132–143. 10.35365/ctjpp.21.2.15

[B19] Guilera G. Pereda N. Paños A. Abad F. J. (2015). Assessing resilience in adolescence: The spanishadaptation of the adolescent resilience questionnaire. *Health Qual. Life Outcomes* 13:100. 10.1186/s12955-015-0259-8 26159814 PMC4498517

[B20] Hagan J. F. Shaw J. S. Duncan P. M. (2017). *Bright Futures Pocket Guide*, 4th Edn. Elk Grove Village, IL: American Academy of Pediatrics, 10.1542/9781610020831

[B21] Hammell K. W. (2004). Dimensions of meaning in the occupations of daily life. *Can. J. OccupTher.* 71 296–305. 10.1177/000841740407100509 15633880

[B22] Hashemi M. Mahmoudzadeh M. (2025). The lived experiences of childhood trauma in war: Has post-traumatic growth occurred? *Eur. J. Psychotraumatol.* 16:2468605. 10.1080/20008066.2025.2468605 40035687 PMC11881655

[B23] Höltge J. Theron L. C. Cowden R. G. Govender K. Maximo S. I. Carranza J. S. et al. (2021). A cross-country network analysis of adolescent resilience. *J. Adolesc. Health* 68 580–588. 10.1016/j.jadohealth.2020.07.010 32919888

[B24] IBM Corp (2022). *IBM SPSS Statistics for Windows. Version 29.0 [computer software].* Armonk, NY: IBM Corp.

[B25] Imms C. Adair B. Keen D. Ullenhag A. Rosenbaum P. Granlund M. (2017). ‘Participation’: Aconceptual framework for understanding outcomes of family-centered services. *Dev. Med. Child Neurol.* 59 983–991. 10.1111/dmcn.13437 28432692

[B26] Kim E. Park J. (2023). Structural relationship among disability acceptance, resilience, and social participation of adolescents with physical disabilities. *Int. J. Disabil. Dev. Educ.* 70 1085–1100. 10.1080/1034912X.2021.1944610

[B27] Kimhi S. Eshel Y. Zysberg L. Hantman S. (2010). Sense of danger and family support as mediators of adolescents’ distress and recovery in the aftermath of war. *J. Loss Trauma* 15 351–369. 10.1080/15325024.2010.491769

[B28] Klochko A. (2024). Impact of war on the mental health of adolescents. *Balt. J. Leg. Soc. Sci.* 1 205–211. 10.30525/2592-8813-2024-1-25

[B29] Lam C. B. McHale S. M. Crouter A. C. (2014). Time with peers from middle childhood to late adolescence: Developmental course and adjustment correlates. *Child Dev.* 85 1677–1693. 10.1111/cdev.12235 24673293 PMC4107039

[B30] Lavie-Pitaro Y. Weintraub N. Golos A. (2025). Adolescent participation characteristics: Development and initial psychometric properties of the Adolescents Participation Questionnaire. *Scand. J. Occup. Ther.* 32:2487467. 10.1080/11038128.2025.2487467 40243200

[B31] Lee J. S. Ahn Y. S. Jeong K. S. Chae J. H. Choi K. S. (2014). Resilience buffers the impact of traumatic events on the development of PTSD symptoms in firefighters. *J. Affect. Disord.* 62 128–133. 10.1016/j.jad.2014.02.031 24767017

[B32] Liebenberg L. Theron L. Sanders J. Munford R. Van Rensburg A. Rothmann S. et al. (2016). Bolstering resilience through teacher-student interaction: Lessons for school psychologists. *Sch. Psychol. Int.* 37 140–154. 10.1177/0143034315614689

[B33] Llistosella M. Goni-Fuste B. Martín-Delgado L. Miranda-Mendizabal A. Franch Martinez B. Pérez-Ventana C. et al. (2023). Effectiveness of resilience-based interventions in schools for adolescents: A systematic review and meta-analysis. *Front. Psychol.* 14:1211113. 10.3389/fpsyg.2023.1211113 37868613 PMC10587685

[B34] Maalouf F. T. Alrojolah L. Ghandour L. Afifi R. Dirani L. A. Barrett P. et al. (2020). Building emotional resilience in youth in Lebanon: A school-based randomized controlled trial of the FRIENDS intervention. *Prev. Sci.* 21 650–660. 10.1007/s11121-020-01123-5 32363411

[B35] Marciano H. Kimhi S. Eshel Y. Adini B. (2024). Resilience and coping during protracted conflict: A comparative analysis of general and evacuees populations. *Isr. J. Health Policy Res.* 13:56. 10.1186/s13584-024-00642-8 39358809 PMC11448295

[B36] McLaughlin P. Kennedy B. Harris A. Hamilton M. Richardson J. Holman-Jones S. (2021). Online and social media resilience in young people in vulnerable contexts. *Vulnerable Child Youth Stud.* 16 178–188. 10.1080/17450128.2020.1849886

[B37] Nizzero A. Cote P. Cramm H. (2017). Occupational disruption: A scoping review. *J. Occup. Sci.* 24 114–127. 10.1080/14427591.2017.1306791

[B38] Oberle E. Ji X. R. Guhn M. Schonert-Reichl K. A. Gadermann A. M. (2019). Benefits of extracurricular participation in early adolescence: Associations with peer belonging and mental health. *J. Youth Adolesc.* 48 2255–2270. 10.1007/s10964-019-01110-2 31440881

[B39] Oliver K. G. Collin P. Burns J. Nicholas J. (2006). Building resilience in young people through meaningful participation. *Aust. e-J. Adv. Ment. Health* 5 34–40. 10.5172/jamh.5.1.34

[B40] Olsson C. A. Bond L. Burns J. M. Vella-Brodrick D. A. Sawyer S. M. (2003). Adolescent resilience: A concept analysis. *J. Adolesc.* 26 1–11. 10.1016/S0140-1971(02)00118-5 12550818

[B41] Osokina O. Silwal S. Bohdanova T. Hodes M. Sourander A. Skokauskas N. (2023). Impact of the Russian invasion on mental health of adolescents in Ukraine. *J. Am. Acad. Child Adolesc. Psychiatry* 62 335–343. 10.1016/j.jaac.2022.07.845 36441074

[B42] Ozbay F. Johnson D. C. Dimoulas E. Morgan C. A. III Charney D. Southwick S. (2007). Social support and resilience to stress: From neurobiology to clinical practice. *Psychiatry* 4 35–40. PMC2921311 20806028

[B43] Rutter M. (1987). Psychosocial resilience and protective mechanisms. *Am. J. Orthopsychiatry* 57 316–331. 10.1111/j.1939-0025.1987.tb03541.x 3303954

[B44] Rutter M. (2006). Implications of resilience concepts for scientific understanding. *Ann. N. Y. Acad. Sci.* 1094 1–12. 10.1196/annals.1376.002 17347337

[B45] Sarid O. Hamama L. Hamama-Raz Y. (2025). Coping, meaning in life, and quality of life during ongoing conflict: Insights from Israeli populations. *Isr. J. Health Policy Res.* 14:1. 10.1186/s13584-024-00665-1 39780281 PMC11715607

[B46] Singh R. Mahato S. Singh B. Bhushal S. Fomani F. K. (2019). Psychometric properties ofAdolescent Resilience Questionnaire among Nepalese adolescents in Lalitpur. *Asian J. Psychiatr.* 45 13–17. 10.1016/j.ajp.2019.08.002 31430691

[B47] Sippel L. M. Pietrzak R. H. Charney D. S. Mayes L. C. Southwick S. M. (2015). How does social support enhance resilience in the trauma-exposed individual? *Ecol. Soc.* 20:10. 10.5751/ES-07832-200410

[B48] Song L. Zhang Z. (2023). “Conceptualisation of Social Support: Definition and Typology,” in *The SAGE Handbook of Social Network Analysis*, 2nd Edn, eds McLevey J. Scott J. Carrington P. J. (Thousand Oaks, CA: Sage), 322–335.

[B49] Stein B. D. Jaycox L. H. Kataoka S. H. Wong M. Tu W. Elliott M. N. et al. (2003). A mental health intervention for schoolchildren exposed to violence: A randomized controlled trial. *JAMA* 290 603–611. 10.1001/jama.290.5.603 12902363

[B50] Syed M. McLean K. C. (2017). “Erikson’s Theory of Psychosocial Development,” in *The SAGE Encyclopedia of Intellectual and Developmental Disorders*, eds Braaten E. Willoughby B. (Thousand Oaks, CA: Sage), 10.31234/osf.io/zf35d

[B51] Theron L. C. (2016). The everyday ways that school ecologies facilitate resilience: Implications for school psychologists. *Sch. Psychol. Int.* 37 87–103. 10.1177/0143034315615937

[B52] Tollit M. McDonald M. Borschmann R. Bennett K. von Sabler M. Patton G. (2015). *Epidemiological Evidence Relating to Resilience and Young People.* Melbourne, VC: Victoria Health Promotion Foundation.

[B53] Too E. K. Chongwo E. Mabrouk A. Abubakar A. (2022). Adolescent connectedness: A scopingreview of available measures and their psychometric properties. *Front. Psychol.* 13:856621. 10.3389/fpsyg.2022.856621 35664205 PMC9159472

[B54] Ungar M. (2018). Systemic resilience. *Ecol. Soc.* 23:34. 10.5751/ES-10385-230434

[B55] Ungar M. Ghazinour M. Richter J. (2013). Annual research review: What is resilience within the social ecology of human development? *J. Child Psychol. Psychiatry* 54 348–366. 10.1111/jcpp.12025 23215898

[B56] Uysal B. Yanik M. Tastekne F. Tuzgen E. Altinisik E. Acarturk C. (2022). Psychological problems and resilience among Syrian adolescents exposed to war. *Eur. J. Trauma Dissociat.* 6:100258. 10.1016/j.ejtd.2022.100258

[B57] Wilcock A. A. (1999). The doris sym memorial lecture: Developing a philosophy of occupationfor health. *Br. J. Occup. Ther.* 62 192–198. 10.1177/030802269906200503

[B58] World Health Organization (2007). *International Classification of Functioning, Disability and Health: Children and Youth Version (ICF-CY).* Geneva: WHO.

[B59] Zimet G. D. Dahlem M. W. Zimet S. G. Farley G. K. (1988). The multidimensional scale for perceived social support. *J. Pers. Assess* 52 30–41. 10.1207/s15327752jpa5201_2

